# Application of IMB model in preventing venous thromboembolism in elderly lung cancer patients

**DOI:** 10.3389/fcvm.2024.1352515

**Published:** 2024-02-16

**Authors:** Mengdan Liu, Xushu Chen, Peng Ma, Zhuoxin Yang, Min Jiang, Min Deng

**Affiliations:** ^1^ School of Nursing, Chengdu Medical College, Chengdu, Sichuan, China; ^2^ Intensive Care Unit, Longquanyi District Hospital of Chengdu University of Traditional Chinese Medicine, Chengdu, Sichuan, China; ^3^ The Publicity Division, Neijiang First People’s Hospital, Neijiang, Sichuan, China

**Keywords:** information-motivation-behavioral skills model, venous thromboembolism, elderly lung cancer, quality of life, incidence rate

## Abstract

**Objective:**

This study aims to explore the effects of the Information-Motivation-Behavioral (IMB) Skills Model on the prevention of Venous Thromboembolism (VTE) in elderly lung cancer patients.

**Methods:**

A convenience sampling method was used to select study participants who were hospitalized for treatment between November 2022 and August 2023 at a tertiary hospital in Neijiang and met the inclusion and exclusion criteria. The control group (*n* = 41) received conventional health education, while the intervention group (*n* = 40) received health education based on the IMB Skills Model over three months. The scores of the Venous Thrombosis Knowledge, Participation in Thrombosis Prevention Willingness and Behavior Questionnaire, and Quality of Life Measurement Scale (QLQ-C30) were compared before the intervention and after three months. After three months of intervention, the hospital satisfaction and VTE incidence rates in both groups were investigated and compared.

**Results:**

After three months of intervention, the scores for the Venous Thrombosis Knowledge, (Participation in Thrombosis Prevention Willingness and Behavior Questionnaire in the intervention group were higher than those in the control group (*P* < 0.05). The QLQ-C30 scores in the intervention group for physical function, role function, emotional function, insomnia, appetite loss, and overall health status were higher than those in the control group (*P* < 0.05). The intervention group rated higher in doctor's professional skills, information provision, accessibility; nurse's professional skills, humanistic care, information provision, accessibility; team communication, services of other personnel, overall satisfaction compared to the control group (*P* < 0.05). The rate of VTE in the intervention group was 2.5%(1/40), and that in the control group was 19.5%(8/41). There was a significant difference (*χ*^2^ = 4.336, *P* = 0.037).

**Conclusion:**

Nursing interventions based on the IMB Skills Model for elderly lung cancer patients can enhance patients’ understanding of venous thrombosis, increase willingness and active participation in thrombosis prevention, improve quality of life, increase hospital satisfaction, and reduce the incidence of VTE.

## 1 Introduction

Primary bronchogenic carcinoma, commonly referred to as lung cancer, is also known as “cancer of the elderly.” It is one of the malignancies with high incidence and mortality rates both in China and globally (1). Elderly lung cancer patients often experience accelerated spread of tumor cells due to various chronic underlying diseases. Poor physical condition and prolonged bed rest can lead to slower venous blood flow or stasis, with a VTE incidence rate of 8.54% (2). Chemotherapy, a common treatment for lung cancer patients, can damage venous vessels (3, 4), thereby controlling cancer while further increasing the risk of concurrent venous thromboembolism (VTE) (5, 6), delaying the rehabilitation process, and increasing the treatment burden for patients (7). There is an urgent need for scientifically effective and personalized health education interventions to improve this situation. The Information-Motivation-Behavioral Skills (IMB) model addresses information, motivation, and behavioral skills to guide patients in establishing positive health behaviors (8). It has been widely used in health education and has yielded favorable results (9–11). Therefore, this study is based on the IMB model to provide health education on the prevention of venous thromboembolism for elderly lung cancer patients, observing its application effects.

## 2 Subjects and methods

### 2.1 Sample subjects

In this study, convenience sampling method was used to select inpatients with chemotherapy for lung cancer who were hospitalized in the oncology department of a tertiary general hospital in Sichuan Province from November 2022 to August 2023.Inclusion criteria: (1) Pathologically or cytologically confirmed primary lung cancer (12); (2) Age ≥60 years (13); (3) Patients informed about the diagnosis; (4) Able to understand the content of the questionnaire and communicate smoothly; (5) A Padua score of ≥4 on the VTE prevention risk assessment; (6) At least two or more chemotherapy cycles received at this hospital. Exclusion criteria: (1) Presence of mental disorders or impaired consciousness; (2) Participation in other studies; (3) Lost to follow-up during the intervention. This study was reviewed and approved by the hospital's medical ethics committee (approval number: 2022-Ethics Approval-10), and all patients signed an informed consent form.

### 2.2 Sample size estimates

The sample size estimation of this study was based on the formula of the mean of the two samples: N1 = N2 = 2[(µ*_ɑ_*+µ*_β_*)/(δ/σ)]^2 ^+ 0.25µ*_ɑ_*^2^, and the scores of VTE prevention knowledge, willingness to participate and behavior questionnaires were used as the calculation indexes, and u*_α_* and u*_β_* were the u-values corresponding to the test level α and type II error probability β respectively, α = 0.05 (two-sided), *β*=0.10 (one-sided), u_0.05/2 _= 1.96, and u_0.1 _= 1.28. σ is the standard deviation of the population (assuming that the standard deviations of the two populations are equal) and δ is the difference between the means of the two populations. According to the results of the pre-experiment, the scores of VTE prevention knowledge, participation intention and behavior in the control group and intervention group were 97.30 ± 8.69, 104.80 ± 9.59, and N1 = N2 = 35 cases were calculated, considering that the patients may be lost to follow-up and dropped out halfway, and the loss rate was calculated at 20%, and finally N1 = N2 = 42 cases were obtained. Since there was 1 death in the control group, 1 death and 1 withdrawal in the intervention group, finally, 41 in the control group and 40 in the intervention group.

### 2.3 Research methods

#### 2.3.1 Control group

Upon admission, the control group was provided with standard health education. During the chemotherapy period, nursing staff provided the patients with VTE prevention brochures for bedside health education once a month, lasting 15–20 min per session, which included the principles of VTE occurrence, its hazards, preventive measures, and treatment methods. For patients during the chemotherapy intermission period, bi-weekly telephone follow-ups were conducted to inquire about the patient's current physical condition and medication usage, providing timely education based on the patient's situation. This included guidance on VTE prevention exercises and reminders for patients to seek medical attention promptly if VTE-related symptoms occurred. Drug prevention: Treatment with plain heparin or LMWH if necessary. The number of VTE occurrences during the intervention was recorded, and patients were guided to follow medical advice for subsequent treatments.

#### 2.3.2 Intervention group

##### 2.3.2.1 Establishment of research team

The research team consisted of one chief nurse in oncology, serving as the team leader responsible for the supervision of the intervention process and management and communication with the department’s patients; one oncology expert and one geriatrics expert responsible for answering disease-related knowledge during the health education development process; one head of the oncology department organized VTE-related knowledge training and assessment for medical and nursing staff; and two responsible nurses, who together with the researcher, were in charge of implementing the intervention plan and data entry.

##### 2.3.2.2 Intervention content

The intervention group received health education based on the IMB skills model in addition to the control group’s standard care. The specific intervention methods are shown in Supplementary Table S1.

### 2.4 Observation indicators and evaluation tools

#### 2.4.1 Venous thrombosis knowledge, participation in thrombosis prevention willingness, and behavior questionnaire

Developed by Yanting Cai and other scholars (14), this questionnaire mainly includes three dimensions: prevention knowledge, willingness to participate, and participatory behavior, with a total of 30 items. Higher scores indicate a higher level of patient knowledge about venous thrombosis, willingness to participate, and active behavior. The questionnaire's Cronbach's alpha coefficient ranges from 0.836 to 0.899, indicating good reliability and validity.

#### 2.4.2 Quality of life measurement scale (QLQ-C30)

Developed by the European Organization for Research and Treatment of Cancer (EORTC) (15), this is a commonly used scale for assessing the quality of life of cancer patients. It consists of 30 items, with items 29 and 30 being seven-grade, scored from 1 to 7 points and considered positive scoring items; the remaining items are four-grade, corresponding to 1–4 points and considered negative scoring items. Higher scores in the functional domains and overall health indicate better function and health status, while higher scores in the symptom domains indicate more severe symptoms. The Cronbach's alpha coefficient for the various dimensions ranges from 0.746 to 0.878, indicating good reliability and validity.

#### 2.4.3 Chinese version of the EORTC in-PATSAT32 hospital satisfaction questionnaire

Translated by Professor Zhiqin Luo and her team (16), this questionnaire is used to survey cancer patients’ satisfaction with medical care, nursing, and hospital services across 14 dimensions. The questionnaire uses a 5-point Likert scale, with higher scores indicating greater patient satisfaction. The Cronbach's alpha coefficient for the various dimensions ranges from 0.86 to 0.96, indicating good reliability and validity.

#### 2.4.4 Venous thromboembolism (VTE) incidence rate in patients

During the study, plasma D-dimer, Doppler ultrasound or angiography for symptoms or signs of suspected deep vein thrombosis/catheter-related thrombosis (17), and CTPA, pulmonary arteriography, MRPA or bedside echocardiography for patients with symptoms or signs of suspected pulmonary thromboembolism (18).Two researchers recorded the occurrence of VTE in patients and compared the incidence rates between the two groups. VTE incidence rate (deep vein thrombosis + pulmonary embolism + catheter-related thrombosis) = number of occurrences/total number of cases included in the study.

### 2.5 Observation indicators

#### 2.5.1 Main outcome

The venous thrombosis knowledge, willingness to participate in thrombosis prevention and behavior scores of the two groups were compared before and after the intervention.

#### 2.5.2 Secondary outcomes

1. The quality of life of the two groups was compared before and after the intervention.
2. The scores of inpatient satisfaction were compared between the two groups after the intervention.
3. The incidence of VTE was compared between the two groups after intervention.

### 2.6 Statistical methods

The data obtained were analyzed using IBM SPSS Statistics 26.0. Categorical data were expressed as frequency and percentage; measurement data that conformed to normal distribution were expressed as mean ± standard deviation, and those that did not conform to normal distribution were expressed as median (interquartile range) M (*P*_25_, *P*_75_). Statistical inference: Measurement data were analyzed using two-sample *t*-tests or rank sum tests; intergroup and intragroup comparisons before and after intervention were performed using paired sample *t*-tests or approximate *t*-tests; categorical data were analyzed using chi-square tests, with the significance level set at *α *= 0.05.

## 3 Results

### 3.1 Comparison of general data between the two groups before the intervention

There was no statistical significance in the comparison of general data between the two groups (*P* > 0.05), indicating comparability. 9.8% (4/41) of the control group received oral prophylactic drug treatment, 34.1% (14/41) received subcutaneous injection of heparin or low molecular weight heparin sodium treatment; 7.5% (3/40) of the intervention group received oral drug treatment, and a total of 32.5% (13/40) received subcutaneous injection of heparin or low molecular weight heparin treatment. The rest did not receive medication for prevention (*χ*^2^ = 0.072, *P* = 0.788), as shown in Supplementary Table S2.

### 3.2 Comparison of venous thrombosis prevention knowledge, willingness, behavior, and total scores before and after intervention between the two groups

There is interesting in showing that all parameters improved, both in the intervention and control group. And after the intervention, the scores for venous thrombosis prevention knowledge, willingness, behavior, and total scores in the intervention group were higher than those in the control group (*P *< 0.05), as shown in Supplementary Table S3.

### 3.3 Comparison of quality of life scores before and after intervention between the two groups

After the intra-group comparison, both groups showed little change in constipation, diarrhea, or financial difficulties(*P > *0.05), the patients in the control group improved in physical function, fatigue, pain and insomnia(*P *< 0.05), and patients in the intervention group were improved effectively in the remaining aspects(*P *< 0.05). After the intervention, intergroup comparisons revealed statistically significant differences between the two groups in scores for physical function, role function, emotional function, insomnia, appetite loss, and overall health status (*P *< 0.05), as shown in Supplementary Table S4.

### 3.4 Comparison of hospital satisfaction after intervention between the two groups

After the intervention, intergroup comparisons revealed statistically significant differences in scores for doctor's professional skills, doctor's information provision, doctor's accessibility, nurse's professional skills, nurse's humanistic care, nurse's information provision, services of other personnel, hospital convenience and overall satisfaction (*P *< 0.05). Among them, the hospital environment scored the lowest (Z = −1.187, *P* = 0.235), the comparison between the two groups is most obvious in the “other personnel service” category (Z = −2.447, *P *= 0.014), as shown in Supplementary Table S5.

### 3.5 Comparison of VTE incidence between the two groups

The rate of VTE in the intervention group was 2.5% (1/40), and that in the control group was 19.5% (8/41). There was a significant difference (*χ*^2^ = 4.336, *P* = 0.037), as shown in Supplementary Table S6.

## 4 Discussion

### 4.1 Health education based on the IMB model can promote changes in VTE prevention knowledge, willingness to participate, and behavior among elderly lung cancer patients undergoing chemotherapy

Globally, public awareness of VTE is generally low (19, 20). Elderly patients, due to various factors, have limited understanding of VTE, further increasing their risk of developing the condition (2, 20–22). Kathryn (23) showed that sources of health knowledge about VTE were primarily the internet (59%), family and friends (47%), magazines and news (18%), and television (17%). When asked about their preferred method of receiving health information on VTE, 87% of participants chose health education. This study implemented health education on VTE prevention based on the IMB model for elderly lung cancer patients, and the results showed that patients in the intervention group significantly improved in terms of VTE prevention knowledge (Z = −3.161, *P *< 0.05), willingness to participate in prevention (Z = −5.820, *P *< 0.05), and preventive behavior (t = 5.932, *P *< 0.05). This indicates that health education based on the IMB model can enhance patients’ self-preventive abilities for VTE more effectively than general health education. Health education for VTE prevention based on the IMB model mainly includes information, motivation, and behavior. Information is a prerequisite; this study, taking into account the clinical characteristics of elderly lung cancer patients, used individualized and group interventions, bedside education, health lectures, and telephone Q&A sessions to provide comprehensive and systematic knowledge of VTE prevention, helping patients to better understand the pathogenesis of VTE and to improve their knowledge of the disease (24, 25). Motivation is the determining factor for behavior change. By organizing patient support groups and medical staff-patient seminars, the study helped patients recognize the importance of VTE prevention and thus stimulated their motivation to establish preventive behaviors (26). The influence of information and motivation on behavior change is mainly realized through behavioral skills. Considering the cultural differences among elderly patients, this study used video guidance and physical demonstrations for interventions and invited family members to learn together to leverage the supervisory role of family members, helping patients establish good and correct exercise habits (27).The results showed that 47.5% (19/40) patients in the intervention group insisted on daily functional exercise, while only 4.9% (2/41) patients in the control group(*χ*^2 ^= 24.815, *P *< 0.05); When limb swelling, pain and other symptoms occurred, 14.6% (6/41) of the control group chose to actively inform the medical staff, while 65% (26/40) of the intervention group(*χ*^2 ^= 22.244, *P *< 0.05), it shows that the IMB model plays a positive role in helping patients establish good behavior habits.

### 4.2 Health education based on the IMB model can improve quality of life in elderly lung cancer patients undergoing chemotherapy

Most elderly lung cancer patients undergoing chemotherapy bear a heavy psychological burden due to long-term disease treatment and severe economic pressures, which affects their quality of life (28–30). After the intervention, the intervention group scored higher than the control group in physical function, role function, emotional function, and overall health status, and lower in insomnia and pain (*P* < 0.05). This indicates that health education based on the IMB skills model can improve the quality of life for elderly lung cancer patients undergoing chemotherapy. The analysis suggests that the health education for VTE prevention based on the IMB model, by guiding patients in functional exercise, not only improves their physical function but also their proactivity (31). Moreover, by conducting support group meetings and face-to-face patient discussions, patients realize the importance of VTE prevention while alleviating stress, enhancing their confidence in coping with the disease, and thus improving their role and emotional functions and overall quality of life (32). Other dimensions such as symptom scales, as well as constipation, diarrhea, and financial difficulties, did not show significant differences after the intervention in both groups, which may be related to disease progression causing increased symptoms and medical expenses (33–35). The IMB skills model, by providing comprehensive and rich information, enables patients to better understand the pathogenesis of VTE and recognize early symptoms, thereby enhancing their understanding of disease knowledge. It emphasizes engaging patients in VTE prevention, enhancing their perception of the risk of VTE, and guiding them to establish behaviors to prevent VTE, which helps to improve self-management abilities and quality of life.

### 4.3 Health education based on the IMB model Can increase hospital satisfaction among elderly lung cancer patients undergoing chemotherapy

Patient satisfaction during hospitalization is an important indicator for evaluating the quality of hospital service management. By analyzing patients’ subjective responses after receiving medical services, the effectiveness of work can be improved, and patient satisfaction can be increased (36). The results of this study show that patient satisfaction in the intervention group who received health education based on the IMB skills model was higher than in the control group.The intervention program not only focused on disease prevention education but also paid attention to the quality of life of patients and the continuity of services outside the hospital during chemotherapy breaks, aligning with the “biopsychosocial” medical model, hence the significant effect in the humanistic care dimension. The results of the study showed that waiting time, hospital convenience, and hospital environment did not exhibit noticeable improvements, likely due to objective factors such as high patient traffic and outdated infrastructure, which cannot be improved through health education alone.

### 4.4 Health education based on the IMB model Can reduce the incidence of VTE in elderly lung cancer patients undergoing chemotherapy

VTE often has insidious onset, with patients commonly exhibiting no noticeable symptoms or presenting with limb swelling and pain, leading to low early diagnosis rates. The results of this study show that the incidence of VTE in the intervention group over three months was 2.5%, which is lower than that reported in the study by Kenmotsu (37). Health education based on the IMB skills model, which focuses not only on VTE disease prevention during inpatient chemotherapy but also consolidates disease-related knowledge through telephone follow-ups with knowledge quizzes during chemotherapy intervals, has improved the capability of patients to self-prevent VTE behaviors by managing risk factors both inside and outside the hospital setting, thereby reducing the incidence of VTE.

## 5 Conclusion

Health education based on the IMB model can facilitate changes in VTE prevention knowledge, willingness to participate in prevention, and behaviors among elderly lung cancer patients undergoing chemotherapy. This contributes to the improvement of patients’ quality of life and hospital satisfaction and reduces the incidence of VTE. However, this study also has some shortcomings: for example, this study only conducted a small-scale intervention study in one department, and its intervention measures may not be suitable for others, so the feasibility of IMB model can be confirmed in a larger intervention study in the future. Besides, in future information provision, more scientific and comprehensive VTE prevention education should be provided to patients, while also enhancing medical staff's knowledge regarding the treatment of VTE. In terms of quality of life, there were no significant improvements in scores for pain and difficulty breathing after the interventions in either group, indicating that future interventions should include pain management (38) and pulmonary respiratory function exercises (39, 40) to comprehensively improve the quality of life for elderly lung cancer patients. Additionally, this study had a small sample size and the subjects were from a single center, therefore the next step should be to expand the sample size and verify the conclusions through multicenter studies.

## Data Availability

The original contributions presented in the study are included in the article/Supplementary Material, further inquiries can be directed to the corresponding author.
