# Supplementary material for: Application of IMB model in preventing venous thromboembolism in elderly lung cancer patients
Source: Front Cardiovasc Med. 2024 Feb 16;11:1352515. doi: 10.3389/fcvm.2024.1352515 (PMC10904599; doi:10.3389/fcvm.2024.1352515)
Supplement: Supplementary file 1 [file Table1.docx]

Supplementary Material

# Supplementary Tables

**Table 1** Intervention Plan Based on the IMB Skills Model

| Information Intervention | Motivation Intervention | Behavioral Intervention |
| --- | --- | --- |
| 1. Invite patients and their families to participate in health lectures twice a month, for a total of six topics: ① Basic knowledge of VTE; ② The harm of VTE; ③ Three-level prevention strategies for VTE; ④ VTE video health education; ⑤ Daily life guidance for VTE prevention; ⑥ Prevention of catheter-related thrombosis.  2. Knowledge quiz: During telephone follow-ups with patients in the chemotherapy intermission period, add a VTE knowledge quiz section. Before learning new content each time, review the knowledge points from the last session and use questioning to revise the content learned. | 1. On the first day of each month, hold a patient exchange meeting, inviting patients diagnosed with VTE to discuss their VTE symptoms and treatment process.  2. On the fifteenth of each month, hold a medical staff-patient symposium to gradually guide patients to recognize the importance of VTE prevention during chemotherapy. Invite patients and their families to discuss their understanding of VTE, allowing patients to feel the care and love of their families and help them better establish VTE prevention behavioral habits. | Combined with information intervention, enter the behavioral skills intervention phase immediately after each bedside education session or health lecture during the hospital stay, lasting 15-20 minutes each time.  1. Video guidance: Provide VTE prevention exercise video guidance based on the theoretical content of the information intervention, using the patient's ability to complete the exercises independently as the standard.  2. Action demonstration: Through standard action demonstrations, enable family members and patients to master the essentials of VTE prevention exercises, quickly correct incorrect movements, and enable patients to better grasp the knowledge of functional exercises for VTE prevention, solidifying the theoretical content of the information intervention. |
