# Supplementary material for: Application of IMB model in preventing venous thromboembolism in elderly lung cancer patients
Source: Front Cardiovasc Med. 2024 Feb 16;11:1352515. doi: 10.3389/fcvm.2024.1352515 (PMC10904599; doi:10.3389/fcvm.2024.1352515)
Supplement: Supplementary file 2 [file Table2.docx]

Supplementary Material

# Supplementary Tables

**Table 2** Comparison of General Data of Patients in Both Groups Before Intervention

| Item |  | Number of Cases | Intervention Group (n=40) Number (%) | Control Group (n=41) Number (%) | χ^2^ | *P* |
| --- | --- | --- | --- | --- | --- | --- |
| Age | 60-69 | 55 | 29(72.5) | 26（63.4） | 1.084 | 0.298 |
|  | 70-79 | 23 | 10(25) | 13（31.7） |  |  |
|  | 80-89 | 2 | 1(2.5) | 1（2.4） |  |  |
|  | 90 and above | 1 | 0（0） | 1（2.4） |  |  |
| Gender | Male | 65 | 32（80） | 33（80.5） | 0.003 | 1.000 |
|  | Female | 16 | 8（20） | 8（19.5） |  |  |
| Educational Level | Elementary school and below | 39 | 21（52.5） | 18（43.9） | 1.369 | 0.242 |
|  | Junior high to high school | 37 | 18（45） | 19（46.3） |  |  |
|  | University and above | 5 | 1（2.5） | 4（9.8） |  |  |
| Occupation Before Retirement | Farmer | 30 | 16（40） | 14（34.1） | 0.294 | 0.588 |
|  | Worker | 19 | 8（20） | 11（26.8） |  |  |
|  | Self-employed | 3 | 3（7.5） | 0（0） |  |  |
|  | State public servant | 12 | 6（15） | 6（14.6） |  |  |
|  | Enterprise or institution employee | 12 | 5（12.5） | 7（17.1） |  |  |
|  | Other | 5 | 2（5） | 3（7.3） |  |  |
| History of Alcohol Consumption | Yes | 58 | 31（77.5） | 27（65.9） | 1.351 | 0.326 |
|  | No | 23 | 9（22.5） | 14（34.1） |  |  |
| History of Smoking | Yes | 60 | 32（80） | 28（68.3） | 1.445 | 0.312 |
|  | No | 21 | 8（20） | 13（31.7） |  |  |
| Family History of VTE | Yes | 8 | 5（12.5） | 3（7.3） | 0.611 | 0.482 |
|  | No | 73 | 35（87.5） | 38（92.7） |  |  |
| Type of Tumor | Squamous cell carcinoma | 24 | 10（25） | 14（34.1） | 2.970 | 0.085 |
|  | Adenocarcinoma | 37 | 17（42.5） | 20（48.8） |  |  |
|  | Large cell carcinoma | 1 | 0（0） | 1（2.4） |  |  |
|  | Small cell carcinoma | 19 | 13（32.5） | 6（14.6） |  |  |
| Tumor Stage | Ⅰ | 3 | 1（2.5） | 2（4.9） | 3.640 | 0.056 |
|  | Ⅱ | 10 | 2（5） | 8（19.5） |  |  |
|  | Ⅲ | 26 | 13（32.5） | 13（31.7） |  |  |
|  | Ⅳ | 42 | 24（60） | 18（43.9） |  |  |
| Indwelling Catheter | Yes | 37 | 21（52.5） | 16（39） | 1.482 | 0.268 |
|  | No | 44 | 19（47.5） | 25（61） |  |  |
| Surgery in the Last 3 Months | Yes | 5 | 3（7.5） | 2（4.9） | 0.240 | 0.675 |
|  | No | 76 | 37（92.5） | 39（95.1） |  |  |
| Drug prevention | Take orally | 7 | 3（7.5） | 4（9.8） | 0.072 | 0.788 |
|  | Subcutaneous injection | 27 | 13（32.5） | 14（34.1） |  |  |
|  | No | 47 | 24（60） | 23（56.1） |  |  |
