# Supplementary material for: Application of IMB model in preventing venous thromboembolism in elderly lung cancer patients
Source: Front Cardiovasc Med. 2024 Feb 16;11:1352515. doi: 10.3389/fcvm.2024.1352515 (PMC10904599; doi:10.3389/fcvm.2024.1352515)
Supplement: Supplementary file 3 [file Table3.docx]

Supplementary Material

# Supplementary Tables

**Table 3** Comparison of Venous Thrombosis Prevention Knowledge, Willingness, Behavior, and Total Scores Before and After Intervention Between the Two Groups (Points)

| Group | Knowledge | |  | Prevention Willingness | |
| --- | --- | --- | --- | --- | --- |
|  | Before Intervention | After Intervention |  | Before Intervention | After Intervention |
| Intervention Group (n=40) | 13（12,16.75） | 34（29,36）* |  | 27（24.25,29） | 36（34,38）* |
| Control Group (n=41) | 13（12,18） | 31（25,33.5）* |  | 26（24,28.5) | 30（28.5,32）* |
| Z*/t* | -0.282^1)^ | -3.161^1)^ |  | -0.532^1)^ | -5.820^1)^ |
| *P* | 0.778 | 0.002 |  | 0.595 | <0.001 |
| Group | Prevention Behavior | |  | Total Score | |
|  | Before Intervention | After Intervention |  | Before Intervention | After Intervention |
| Intervention Group (n=40) | 30.83±4.84 | 42.95±4.14* |  | 72（66，80.5） | 114(103.25,117.75)* |
| Control Group (n=41) | 31.24±5.47 | 38±3.34* |  | 72（68，80.5） | 99（89.5，104）* |
| Z*/t* | -0.365^2)^ | 5.932^2)^ |  | -0.246^1)^ | -5.227^1)^ |
| *P* | 0.716 | <0.001 |  | 0.806 | <0.001 |

Note: 1) Z value 2) t value; * Compared with the same group before intervention, P<0.05
