# Supplementary material for: Application of IMB model in preventing venous thromboembolism in elderly lung cancer patients
Source: Front Cardiovasc Med. 2024 Feb 16;11:1352515. doi: 10.3389/fcvm.2024.1352515 (PMC10904599; doi:10.3389/fcvm.2024.1352515)
Supplement: Supplementary file 4 [file Table4.docx]

Supplementary Material

# Supplementary Tables

**Table 4 Comparison of Quality of Life Between the Two Groups ($\overline{x}$±s)**

| Group | Physical Function | |  | Role Function | |
| --- | --- | --- | --- | --- | --- |
|  | Before Intervention | After Intervention |  | Before Intervention | After Intervention |
| Intervention Group (n=40) | 80(53.33,93.33) | 83.34(68.34,93.33)* |  | 66.67(66.67,100) | 100(66.67,100)* |
| Control Group (n=41) | 80(66.67,86.67) | 66.67（60，86.67）* |  | 83.33(66.67,100) | 83.33(66.67,83.33) |
| Ｚ | -0.585 | -2.505 |  | -0.713 | -2.834 |
| *P* | 0.558 | 0.012 |  | 0.476 | 0.005 |
| Group | Emotional Function | |  | Cognitive Function | |
|  | Before Intervention | After Intervention |  | Before Intervention | After Intervention |
| Intervention Group (n=40) | 83.33(75,100) | 100(91.67,100)* |  | 83.33(66.67,100) | 83.33(83.33,100)* |
| Control Group (n=41) | 91.67(75,100) | 91.67(75,100) |  | 83.33(88.33,100) | 83.33(66.67,100) |
| Ｚ | -0.847 | -2.172 |  | -1.112 | -0.807 |
| *P* | 0.397 | 0.03 |  | 0.266 | 0.42 |
| Group | Social Function | |  | Fatigue | |
|  | Before Intervention | After Intervention |  | Before Intervention | After Intervention |
| Intervention Group (n=40) | 83.33(66.67,100) | 100(70.84,100)* |  | 33.33(22.22,44.44) | 11.11(2.78,22.22)* |
| Control Group (n=41) | 83.33(66.67,100) | 100(66.67,100) |  | 22.22(16.67,44.44) | 11.11(11.11,33.33)* |
| Ｚ | -0.344 | -1.194 |  | -0.409 | -0.457 |
| *P* | 0.731 | 0.232 |  | 0.683 | 0.648 |
| Group | Nausea and Vomiting | |  | Pain | |
|  | Before Intervention | After Intervention |  | Before Intervention | After Intervention |
| Intervention Group (n=40) | 0(0,33.33) | 0(0,16.67)* |  | 16.67(0,33.33) | 0(0,16.67)* |
| Control Group (n=41) | 0(0,16.67) | 0(0,0) |  | 16.67（0，33.33） | 0(0,16.67)* |
| Ｚ | -0.522 | -0.994 |  | -0.135 | -0.634 |
| *P* | 0.601 | 0.32 |  | 0.893 | 0.526 |
| Group | Dyspnea | |  | Insomnia | |
|  | Before Intervention | After Intervention |  | Before Intervention | After Intervention |
| Intervention Group (n=40) | 33.33(0,33.33) | 0(0,33.33)* |  | 33.33(33.33,66.67) | 0(0,33.33)* |
| Control Group (n=41) | 33.33(0,33.33) | 0(0,33.33) |  | 33.33(33.33，50) | 33.33(0,33.33)* |
| Ｚ | -0.026 | -1.64 |  | -0.515 | -2.251 |
| *P* | 0.979 | 0.101 |  | 0.606 | 0.024 |
| Group | Appetite Decrease | |  | Constipation | |
|  | Before Intervention | After Intervention |  | Before Intervention | After Intervention |
| Intervention Group (n=40) | 33.33(0,66.67) | 0(0,33.33)* |  | 0(0,33.33) | 0(0,33.33) |
| Control Group (n=41) | 33.33(33.33,33.33) | 33.33(0,33.33) |  | 0(0,33.33) | 0(0,33.33) |
| Ｚ | -0.061 | -2.041 |  | -0.541 | -0.53 |
| *P* | 0.951 | 0.041 |  | 0.588 | 0.596 |
| Group | Diarrhea | |  | Financial Difficulties | |
|  | Before Intervention | After Intervention |  | Before Intervention | After Intervention |
| Intervention Group (n=40) | 0(0,0) | 0(0,0) |  | 33.33(33.33,66.67) | 33.33(33.33,66.67) |
| Control Group (n=41) | 0(0,0) | 0(0,0) |  | 33.33(33.33,66.67) | 33.33(33.33,66.67) |
| Ｚ | -0.467 | -0.923 |  | -0.384 | -0.473 |
| *P* | 0.64 | 0.356 |  | 0.701 | 0.636 |
| Group | Overall Health Status | | | | |
|  | Before Intervention | |  | After Intervention | |
| Intervention Group (n=40) | 54.17(41.67,66.67) | |  | 75(52.08,81.25)* | |
| Control Group (n=41) | 50(45.84,62.5) | |  | 58.33(41.67,75) | |
| Ｚ | -0.029 | |  | -2.948 | |
| *P* | 0.977 | |  | 0.003 | |

Note: * Compared with the same group before intervention, *P*<0.05
