# Supplementary material for: Application of IMB model in preventing venous thromboembolism in elderly lung cancer patients
Source: Front Cardiovasc Med. 2024 Feb 16;11:1352515. doi: 10.3389/fcvm.2024.1352515 (PMC10904599; doi:10.3389/fcvm.2024.1352515)
Supplement: Supplementary file 5 [file Table5.docx]

Supplementary Material

# Supplementary Tables

**Table 5** Comparison of Hospital Satisfaction After Intervention Between the Two Groups

| Item | Intervention Group | Control Group | ***Ｚ*** | *P* |
| --- | --- | --- | --- | --- |
| Doctor's Professional Skills | 87.5(75,100) | 75(66.67,91.67) | -2.299 | 0.022* |
| Doctor's Humanistic Care | 91.67(75,100) | 83.33(66.67,100) | -1.576 | 0.115 |
| Doctor's Information Provision | 91.67(75,100) | 83.33(70.84,100) | -2.019 | 0.043* |
| Doctor's Accessibility | 100(87.5,100) | 87.5(75,100) | -2.231 | 0.026* |
| Nurse's Professional Skills | 91.67(75,100) | 75(70.84,95.84) | -2.028 | 0.043* |
| Nurse's Humanistic Care | 91.67(75,100) | 75(70.84,100) | -2.012 | 0.044* |
| Nurse's Information Provision | 91.67(75,100) | 83.33(66.67,100) | -2.001 | 0.045* |
| Nurse's Accessibility | 87.5(75,100) | 87.5(75,100) | -1.205 | 0.228 |
| Team Communication | 75(75,100) | 75(75,100) | -1.016 | 0.31 |
| Services of Other Personnel | 91.67(75,100) | 75(58.33,91.67) | -2.447 | 0.014* |
| Waiting Time | 87.5(75,100) | 75(62.5,93.75) | -1.463 | 0.143 |
| Hospital Convenience | 93.75(75,100) | 75(56.25,100) | -2.065 | 0.039* |
| Hospital Environment | 75(50,75) | 50(50,75) | -1.187 | 0.235 |
| Overall Satisfaction | 75(75,100) | 75(50,75) | -2.44 | 0.015* |

Note: * Compared with the control group, *P*<0.05
