# Supplementary material for: Application of IMB model in preventing venous thromboembolism in elderly lung cancer patients
Source: Front Cardiovasc Med. 2024 Feb 16;11:1352515. doi: 10.3389/fcvm.2024.1352515 (PMC10904599; doi:10.3389/fcvm.2024.1352515)
Supplement: Supplementary file 6 [file Table6.docx]

Supplementary Material

# Supplementary Tables

**Table 6** Comparison of VTE Incidence Between the Two Groups

| Group | Lower Limb Venous Thrombosis | Pulmonary Embolism | Catheter-Related Thrombosis | Total [Cases (%)] |
| --- | --- | --- | --- | --- |
| Intervention Group (n=40) | 0 | 0 | 1 | 1（2.5） |
| Control Group (n=41) | 2 | 1 | 5 | 8（19.5） |
| *χ^2^* |  |  |  | 4.336 |
| *Ｐ* |  |  |  | 0.037 |
